# Evaluation of the Metabolic Activity of the Infiltration and Proliferation Areas of Hepatic Alveolar Echinococcosis in Rats Using Contrast-Enhanced Ultrasound

**DOI:** 10.4269/ajtmh.22-0348

**Published:** 2022-10-31

**Authors:** Xiaorong Wang, Lingfei Yang, Lu Chen, Tao Song

**Affiliations:** Department of Ultrasound, The First Affiliated Hospital of Xinjiang Medical University, Urumqi, People’s Republic of China

## Abstract

This study evaluated the value of contrast-enhanced ultrasound (CEUS) in assessing the metabolic activity of infiltration and proliferation areas of hepatic alveolar echinococcosis (HAE) in rats. CEUS was performed on Wistar rats with HAE. The average grayscale value of the HAE lesion in peripheral infiltration and proliferation areas (PIPAs) and the adjacent normal liver tissue was analyzed quantitatively. Contrast imaging was classified as highly increased enhancement, moderately increased enhancement, and equal or decreased enhancement. Microvessel density (MVD) in the PIPAs was classified as strongly positive, moderately positive, and weakly positive. The metabolic activity of HAE in the PIPAs was classified as high activity, moderate activity, and low activity according to the MVD classification results. The kappa test was combined with the metabolic activity level of the PIPAs to analyze the consistency of CEUS intensity and MVD. CEUS can score the metabolic activity of the infiltration and proliferation areas around HAE lesions, and provides a basis for clinical treatment and follow-up visits. CEUS could be used as a more economical and effective imaging option for evaluating the metabolic activity of HAE lesions.

## INTRODUCTION

Hepatic alveolar echinococcosis (HAE) is considered to be one of the most dangerous parasitic zoonoses in the northern hemisphere.^1^ Clinically, HAE behaves like a malignant tumor, and the prognosis is generally poor. HAE is a serious disease with a > 90% mortality rate in untreated patients.^2^ It has been proposed that the area around the HAE lesion has aggregations of new blood vessels and immune cells, which serves as the basis for the alveolar hydatid to survive and infiltrate the surrounding area.^3^^,^^4^

[^18^F] fluorodeoxyglucose position emission tomography–computed tomography ([^18^F] FDG PET-CT) has been known as an imaging tool for metabolic activity evaluation in HAE. Other imaging methods such as magnetic resonance proton spectroscopy, diffusion imaging, and CT perfusion imaging can also be used for evaluating HAE blood supply and metabolic characteristics.^5^ However, these imaging methods have limited clinical application because of their high cost and radiation exposure. Ehrhardt et al^6^ compared contrast-enhanced ultrasound (CEUS) and [^18^F] FDG PET-CT in 17 patients with HAE. They found that CEUS had a high correlation with FDG PET in determining the metabolic activity of HAE, and that the sensitivity of CEUS was greater than that of FDG PET.^6^ The CEUS manifestation of HAE is presented as a “black hole” center without enhancement and with a “border” enhancement edge. Moreover, there are abundant microvessels in the area with the enhanced edge, which is the active infiltration and proliferation area of HAE.^7^^,^^8^

In this study, the ratio of grayscale contrast intensity between the edge of the rat HAE lesion and the surrounding normal liver was evaluated and graded. Microvessel density (MVD) in the proliferation zone of the edge of the HAE lesion was determined according to the number of stained positive cells. The relationship between these two evaluations was analyzed. Our findings may provide a simple, noninvasive, and nonradiative method for clinical assessment of HAE metabolic activity.

## MATERIALS AND METHODS

### Animals.

This study was approved by the Animal Ethics Committee of our university. Twenty-three Wistar rats (male; 1–3 months old; average body weight, ∼ 200 g) were obtained from the Animal Experimental Center of Xinjiang Medical University, Urumqi, People’s Republic of China. The Mongolian gerbils were obtained from the Laboratory Animal Research Department of the Medical Research Center of The First Affiliated Hospital of Xinjiang Medical College.

### HAE model establishment.

Under aseptic conditions, the Mongolian gerbils were killed by cervical dislocation. Alveolar hydatid tissue was obtained from the fresh liver of gerbils. After removing the necrotic tissue in the central area, tissue homogenate was made, rinsed, and precipitated with saline three times. Then, a 20% concentration of protoscoleces suspension (20,000 protoscoleces/mL) was made after adding saline. Penicillin and streptomycin injections (250 U/mL) were added to the protoscoleces suspension to inoculate the rats. A mixture of 4 mL of anesthetic drug (ketamine solution), 4 mL of anesthesia adjuvant diazepam, and 1 mL of atropine sulfate was diluted with normal saline to 20 mL, and was injected intraperitoneally into rats at a level of 0.75 mL/100 g body weight. After complete anesthesia was induced, all rats were disinfected locally with iodophor, and then 0.2 mL of the *Echinococcus multilocularis* protoscoleces suspension was infused into the liver of the rats after laparotomy. Three months after the inoculation, all rats were subjected to CEUS examination.

### CEUS examination and image analysis.

The Acuson Sequoia512 Ultrasound machine and the 15L8w transducer (8–15 MHz) from Siemens (Munich, Germany) were used for the ultrasound examinations. B-scan ultrasonography of the liver was first performed in all rats. The location, size, shape, boundary, internal echo, and blood flow of the lesion were recorded in detail. For CEUS parameters, the probe frequency was set automatically at 7 MHz; the mechanical index was 0.08 to 0.11. SonoVue^®^ (Bracco Imaging, Milan, Italy) was used as a contrast agent. For angiography, the contrast agent was injected rapidly into the rats through the tail vein at 0.1 mL/injection, followed by an injection of 1 mL saline. The rats were subjected to continuous observation for 5 minutes after angiography. The enhanced mode was used and the images were collected. The duration of each CEUS phase was 2 to 4 seconds in the arterial phase (mean value, 2.9 ± 0.7 seconds), was 3 to 8 seconds in the portal vein phase (mean value, 5.5 ± 2.4 seconds), and was 180 to 300 seconds in the delayed phase (mean value, 233.5 ± 48.6 seconds) based on the angiographic results. Using Image J 1.54 e (National Institutes of Health, Bethesda, MD), the region of interest of three to five annular enhancement bands and adjacent normal liver tissue in the contrast image was selected by avoiding the branches of large blood vessels. The HAE lesions and adjacent areas were highlighted. The software calculated automatically the grayscale values of the infiltration and proliferation area of the HAE lesion and the adjacent normal liver tissue. Using an adjustment method reported by Li et al,^9^ the ratio of the average grayscale values of the HAE lesion to those of adjacent normal liver tissue was calculated. A ratio ≥ 1.1 was defined as an increased enhancement, < 0.9 was defined as a decreased enhancement, and between 0.9 and 1.1 was defined as an equal enhancement.

### Histological analysis.

After CEUS, the rats were killed by cervical dislocation. Liver tissue was collected from areas in line with the corresponding layers of two-dimensional ultrasound and CEUS images. Approximately two to four pieces of tissues at different areas were acquired, which contained the HAE lesion and the surrounding normal liver tissue. The tissues were embedded in paraffin and prepared as 4-μm sections that were then subjected to hematoxylin–eosin staining and immunohistochemical staining. All staining procedures were performed according to standard histological procedures. Phosphate buffered saline, instead of a primary antibody, was used as the negative control. The immunohistochemistry method included a two-step procedure. CD34 was used as a biomarker to represent MVD, with a dilution of 1:100. The CD34 expression was regarded positive in the cytoplasm of vascular endothelial cells when there were pale-yellow, brownish yellow, or tan particles. The MVD score was counted by two trained physicians at different time points using the double-blind method.^10^ Five fields were selected randomly under low magnification (×100), and the number of positive cells was counted under high magnification (×400). The average values of cell counts were used with less than 5% differences. According to the method reported by Zhong et al,^11^ the positively stained cells were divided as follows: > 50%, strongly positive (+++); 11% to 50%, moderately positive (++); and 1% to 10%, weakly positive (+).

### Metabolic activity scoring category of the infiltration and proliferation areas of HAE.

Lesions were scored based on the results of positively stained cells on immunohistochemistry in the peripheral infiltration and proliferation areas of HAE. Immunohistochemical scoring was used as a standard to classify the metabolic activity of the region: high activity of the strongly positive staining was scored as high activity; moderately positive staining, moderate activity; and weakly positive staining, low activity.

### Statistical analysis.

SPSS version 20.0 for Windows statistical software (IBM, Armonk, NY) was used for data analysis. *P* < 0.05 (two sided) was considered statistically significant. The kappa correlation test was used to analyze the contrast enhancement intensity and MVD. Kappa value evaluation criteria were κ ≤ 0.4, poor correlation; 0.4 < κ < 0.75, moderate correlation; and κ ≥ 0.75, good correlation.

## RESULTS

### Results of traditional ultrasound, CEUS, and image analysis.

**Figure 1.**
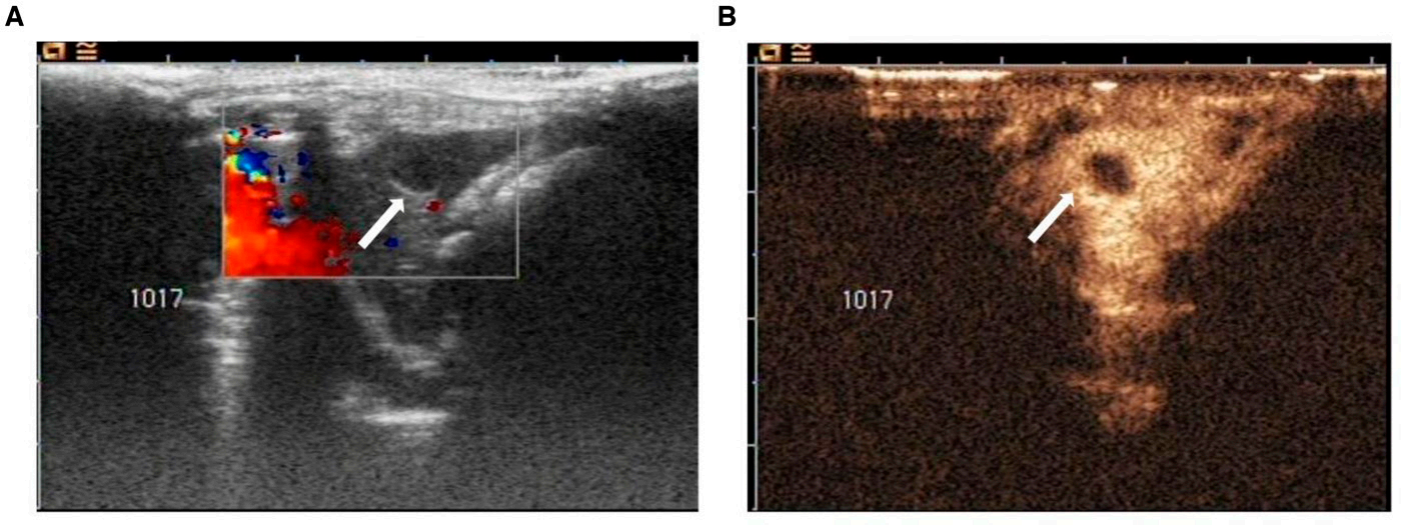
Ultrasound analysis. (**A**) Color Doppler ultrasound images of the hepatic alveolar echinococcosis lesion in a rat liver. The interior region of the lesion shows a round echo signal surrounded by a mid-high echo (white arrow). No significant blood flow signals were detected within the lesion, whereas punctate blood flow signals were visible around the lesion. (**B**) Contrast-enhanced ultrasound characterization of a rat hepatic alveolar echinococcosis lesion. No contrast agent was perfused within the lesion, and the surrounding area shows an annular enhanced signal (white arrow).

Twenty-seven HAE lesions and 117 paraffin-embedded specimens were obtained from 23 infected rats. Using two-dimensional ultrasound, the normal liver tissue of rats showed a uniformly distributed punctate medium echo, and the internal vascular anatomic structure was clearly visible. The HAE lesions showed spherical, spherical-like, or irregular echoes. The peripheral region of HAE was dominated by medium-to-high echoes, some of which were honeycomb shaped, with low echo or no echo divided by the mid-to-high echo. Color Doppler ultrasound showed that no obvious blood flow signals were detected in the 27 lesions. Twenty-four lesions showed peripheral punctate blood flow signals (Figure 1A); no obvious blood flow signals were observed around the other three lesions. The signal enhancement pattern of the 27 HAE lesions showed “fast appear and slow out” mode, and 25 lesions showed peripheral annular enhancement (Figure 1B), and there were filling defects in the annular enhancement zone around the two lesions. There was no significant enhancement in the internal HAE lesions.

**Figure 2.**
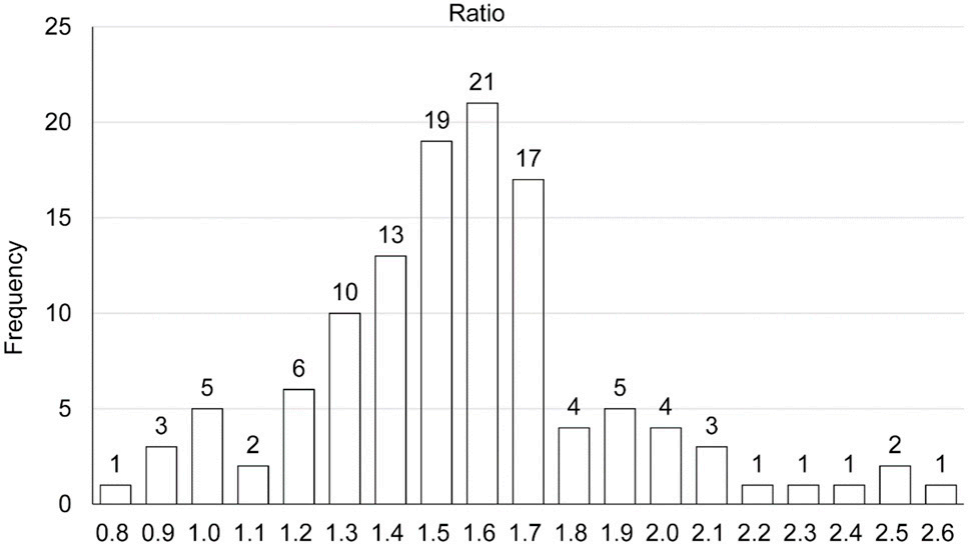
Chart of the frequency distribution of the ratio of the average grayscale values in the infiltration and proliferation areas of the hepatic alveolar echinococcosis lesion to that of the adjacent normal liver tissue.

A total of 119 mean grayscale values were obtained. The grayscale ratio of the peripheral annular enhancement zone to that of adjacent healthy liver tissue of HAE lesions was obtained by quantitative analyses. The maximum ratio was 2.6, the minimum ratio was 0.8, and the mean value was 1.56 ± 0.33 (Figure 2). Approximately 92.4% (110 of 119) showed increased enhancement, 4.2% (5 of 119) showed equal enhancement, and 3.4% (4 of 119) showed decreased enhancement. Furthermore, the increased enhancement group could be divided into two subgroups according to our results—that is, the ratio was defined as highly increased enhancement (18.5%, 22 of 119) at ≥ 1.8 and as moderately increased enhancement (73.9%, 88 of 119) at a value between 1.0 and 1.8.

### Histological analysis of infiltration and proliferation areas of HAE lesions.

**Figure 3.**
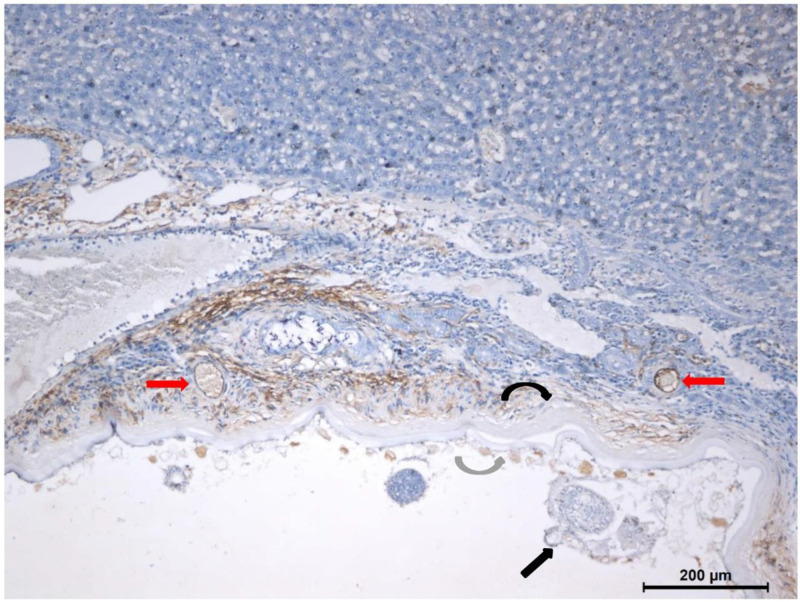
Immunohistochemistry results (×100 magnification) of hepatic alveolar echinococcosis in rats. The laminated layer (black curved arrow) and germinal layer of alveolar larvae were clear (gray curved arrow). The germinal sac and budding structure were observed on the germinal layer. There were different numbers of protoscoleces (black straight arrow) in the germinal layer. The liver tissue adjacent to the lesion was irregular, and many small, new blood vessels were observed around the lesion (red arrows). Scale bar: 200 μm.

The results of immunohistochemistry showed that the structure of the bulbs was relatively normal, with a clear stratum corneum and germinal layer. A germinal sac and budding structure were noted on the germinal layer, along with protocercaria in the germinal sac. The liver tissue adjacent to the lesion was irregular, and new blood vessels were observed around the lesion (Figure 3).

**Figure 4.**
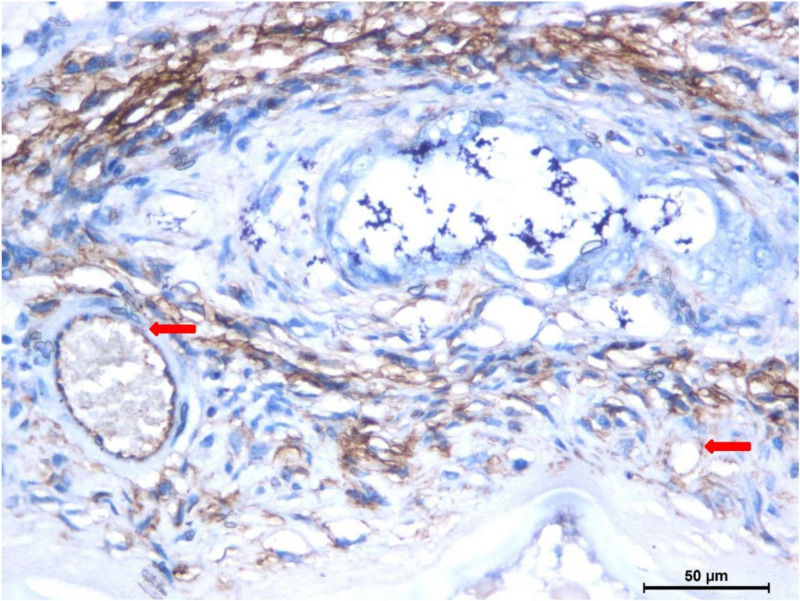
Immunohistochemistry results (×400 magnification). The antigenic marker CD34 was barely expressed in healthy liver tissue, but was highly expressed in the small newborn vessels (red arrows) of the infiltration and proliferation areas of the hepatic alveolar echinococcosis lesions. Scale bar: 50 μm.

The results of immunohistochemistry showed that the antigen marker CD34 was barely expressed in normal liver tissue, and was highly expressed in the infiltration and proliferation areas around the HAE lesions (Figure 4). The positive expression percentage of MVD in the infiltration and proliferative zones around HAE lesions was 100.0% (119 of 119), and 25.2% (30 of 119) were strongly positive, 68.1% (81 of 119) were moderately positive, and 6.7% (8 of 119) were weakly positive.

### Biologic activity of infiltration and proliferation areas of HAE lesions.

According to the MVD scoring results of infiltration and proliferation areas of HAE lesions, 25.2% (30 of 119) had high biological activity, 68.1% (81 of 119) had moderate activity, and 6.7% (8 of 119) had low activity.

### Consistency analysis of CEUS signal enhancement and MVD diagnosis.

The kappa correlation test results of the average grayscale value ratio and the number of positively stained cells showed moderate consistency (κ = 0.641, *P* < 0.05). The results indicate that the two methods show moderate consistency for the diagnosis of the metabolic activity of the infiltration and proliferation areas of HAE lesions (Table 1).

**Table 1:** Consistency of metabolic activity of HAE by CEUS imaging and MVD levels (*N* = 119)

CEUS	MVD (*n*)			Total (*n*)
	Strongly positive	Moderately positive	Weakly positive	
Highly increased enhancement	18	4	0	22
Moderately increased enhancement	12	75	1	88
Equal or decreased enhancement	0	2	7	9
Total	30	81	8	119

CEUS = contrast-enhanced ultrasound; HAE = hepatic alveolar echinococcosis; MVD = microvessel density.

Kappa correlation test: κ = 0.641, *P* < 0.05.

## DISCUSSION

HAE is characterized by infiltration and proliferation of the larvae of *Echinococcus multilocularis* in the liver. Patients with HAE generally have mild symptoms during the early stage, but experience metastasis, secondary infection, and other cachexia at advanced stages. HAE cannot be radically resected and has a poor prognosis. The mortality rate of patients with HAE without proper treatment is nearly 90% within 10 to 15 years after initial diagnosis.^12^ Radical surgery followed by 2 years of benzimidazoles (BMZs) administration is the standard treatment aiming at a cure. However, the percentage of patients who accept radical surgery is less than 30%, and the majority of patients need long-term treatment with BMZs.^13^^,^^14^ Therefore, the survival status and activity of HAE lesions may serve as an important factor in determining the treatment plan.

The metacestodes propagate asexually, like a tumor, first by buds and then by vesicles, eventually leading to bile duct and hepatic vessel obstruction and organ dysfunction. From the very beginning of larval proliferation, the immune response of the host is characterized by the homing of cells to the liver—such as macrophages, lymphocytes, fibroblasts, and myofibroblasts—at the contact of the laminated layer, which displays a “granulomatous” structure.^5^ The periparasitic cell infiltrate is usually located at the periphery of the lesions, in areas with active proliferation of the metacestode. The cytokines secreted by the alveolar hydatid can promote microvascular growth in peripheral granulation tissue.^3^ New vessels along with immune cell homing are present in this granulomatous part of the lesion. Necrotic areas, conversely, are usually located in the most inactive part of the lesion.^5^

[^18^F] FDG PET-CT is currently well recognized as a noninvasive detection tool for evaluating the metabolic activity of alveolar echinococcosis lesions. Usually, patients with HAE undergo [^18^F] FDG PET-CT, which can be used to assess the biologic activity of HAE and efficacy, before receiving treatment. No FDG uptake around the alveolar echinococcosis lesion is often recommended as a criterion for stopping treatment.^5^ However, PET-CT is radioactive and expensive. In particular, the herders in remote areas account for the majority of patients with HAE, which further restricts the clinical application of PET-CT. With the development of ultrasound imaging technology, CEUS has been applied increasingly when evaluating microcirculation. Schwarze et al^15^ suggested that CEUS imaging could be regarded as an easily accessible tool that can describe hypervascularization as a sonomorphological correlate for active perilesional inflammation of echinococcal manifestations. Wa et al^16^ reported 95.4% of the 60 HAE lesions were detected with enhancement on CEUS, which correlates with active texture or an inflammatory reaction belt surrounding the lesion. Our previous study^17^ compared CEUS and [^18^F] FDG PET-CT in 39 HAE lesions of 36 patients and evaluated the mean grayscale ratio of the infiltrating zone of a lesion to the surrounding normal liver tissue by CEUS and the maximum standardized uptake value of [^18^F] FDG PET-CT. We found consistence between CEUS and [^18^F] FDG PET-CT in 87.2% lesions. We also showed previously^7^ that peripheral infiltration and proliferation areas of HAE had abundant blood supply. There is a positive correlation between the signal intensity of CEUS images in the peripheral proliferation zone of HAE and the MVD scores by immunohistochemical staining.^7^ The greater the signal enhancement of the infiltration and proliferation areas of the HAE lesions, the higher the MVD scores.

In the current study, 27 HAE lesions in 23 rats showed “fast appear and slow out” mode, including 25 lesions with annular enhancement in the early arterial phase, and another two lesions that presented a filling defect in the annular enhancement areas of HAE. The ratio of the average grayscale values of the HAE lesion to those of adjacent normal liver tissue was used to evaluate quantitatively the difference in the amount of contrast agent perfusion between the HAE marginal infiltration and proliferation zone and the surrounding normal liver parenchyma. This ratio ranged from 0.8 to 2.6 (mean, 1.56 ± 0.33), indicating there is a wide range of differences in the number of new blood vessels around the HAE lesion.

The peripheral infiltration and proliferation areas of HAE lesions are rich in micro blood vessels. The CD34 marker is also highly expressed in the infiltration and proliferation areas of the HAE lesions, and is rarely expressed in the adjacent healthy liver tissue. SonoVue^®^ is a contrast agent solely distributed in blood circulation. Therefore, the “fast-entry” blood vessels observed in the arterial phase are the newly proliferating blood vessels in the infiltration and proliferation zone around the HAE lesion. There are obviously more new blood vessels in the infiltration and proliferation zone at the edge of the HAE lesion than in the surrounding normal liver tissue. This is the basis for the survival and proliferation of active HAE lesions. The new blood vessels can provide nutrients continuously for the lesion to multiply and expand. The more blood vessels, the more nourishment of HAE, and the stronger the invasion and metastatic ability of the lesion.

MVD is an important indicator of invasion and metastasis of malignancies, which is associated significantly with prognosis in cancer patients.^18^ It has also been shown that the higher MVD is related to higher invasion and metastasis.^19^ In our study, the MVD value of the marginal zone of different HAE lesions was also quite different, which reflects the difference in the biologic activity of the marginal zone of different HAE lesions. The MVD value in the HAE infiltration and proliferation zone correlates positively with its metabolic activity. The greater the MVD, the faster HAE will grow in the liver. Quantitative ultrasound signal enhancement analysis in conjunction with a high expression of MVD in the infiltration and proliferation area demonstrated histopathological evidence of ultrasound annular signal enhancement of HAE lesions. The kappa correlation test results of the average grayscale value ratio to the number of positively stained cells showed moderate consistency (κ = 0.641, *P* < 0.05). Thus, the metabolic level of the infiltration and proliferation area of the HAE lesions could be evaluated using CEUS.

We concluded that CEUS could be used as a more economical and effective imaging option for evaluating the metabolic activity of HAE lesions. For patients with HAE who need to receive medications such as BMZs, CEUS is expected to be a powerful supplement to the evaluation of treatment efficacy. However, our study did not evaluate the role of CEUS in evaluating treatment efficacy of rat HAE lesions. Further studies will be carried out in the future.

## References

[1] Baumann S Shi R Liu W Bao H Schmidberger J Kratzer W Li W, Interdisciplinary Echinococcosis Working Group Ulm , 2019. Worldwide literature on epidemiology of human alveolar echinococcosis: a systematic review of research published in the twenty-first century. Infection 47: 703–727. Erratum in: *Infection 2022;50*:287–288. 31147846 10.1007/s15010-019-01325-2 PMC8505309

[2] Kamiyama T, 2020. Recent advances in surgical strategies for alveolar echinococcosis of the liver. Surg Today 50: 1360–1367. 31768657 10.1007/s00595-019-01922-6

[3] Yibulayin A Li XH Qin YD Jia XY Zhang QZ Li YB, 2018. Biological characteristics of 18F-FDG PET/CT imaging of cerebral alveolar echinococcosis. Medicine (Baltimore) 97: e11801. 30278480 10.1097/MD.0000000000011801 PMC6181521

[4] Tao S Qin Z Hao W Yongquan L Lanhui Y Lei Y, 2011. Usefulness of gray-scale contrast-enhanced ultrasonography (SonoVue^®^) in diagnosing hepatic alveolar echinococcosis. Ultrasound Med Biol 37: 1024–1028. 21640477 10.1016/j.ultrasmedbio.2011.04.014

[5] Liu W Delabrousse É Blagosklonov O Wang J Zeng H Jiang Y Wang J Qin Y Vuitton DA Wen H. 2014. Innovation in hepatic alveolar echinococcosis imaging: best use of old tools, and necessary evaluation of new ones. Parasite 21: 74. doi:10.1051/parasite/2014072. Epub 2014 Dec 23. PMID: 25531446; PMCID: PMC4273719. 25531446 PMC4273719

[6] Ehrhardt AR Reuter S Buck AK Haenle MM Mason RA Gabelmann A Kern P Kratzer W, 2007. Assessment of disease activity in alveolar echinococcosis: a comparison of contrast enhanced ultrasound, three-phase helical CT and [(18)F] fluorodeoxyglucose positron emission tomography. Abdom Imaging 32: 730–736. 17285403 10.1007/s00261-006-9173-1

[7] Song T Li HT Yang LF Yao LH Wen H, 2014. Contrast-enhanced ultrasonography of hepatic alveolar echinococcosis in rats: the correlation of imaging features and histologic microvascular density. Chin J Parasitol Parasit Dis 32: 200–204. 25223055

[8] Schweizer M Schmidberger J Schlingeloff P Kratzer W, 2022. Contrast-enhanced ultrasound (CEUS) in patients with metastasis-like hepatic alveolar echinococcosis: a cohort study. J Ultrasound. 2022 May 21. doi:10.1007/s40477-022-00688-x. Epub ahead of print. PMID: 35597873. PMC10063733 35597873

[9] Li R Hua X Zhang P, 2007. Arterial phase enhancement of liver metastatic carcinoma: comparison of low mechanical index contrast-enhanced ultrasonography with contrast-enhanced CT. Chin J Ultrasound Med 23: 602–604.

[10] Kratzer W Güthle M Dobler F Seufferlein T Graeter T Schmidberger J Barth TF Klaus J, 2022. Comparison of superb microvascular imaging (SMI) quantified with ImageJ to quantified contrast-enhanced ultrasound (qCEUS) in liver metastases: a pilot study. Quant Imaging Med Surg 12: 1762–1774. 35284256 10.21037/qims-21-383 PMC8899953

[11] Zhong H De Marzo AM Laughner E Lim M Hilton DA Zagzag D Buechler P Isaacs WB Semenza GL Simons JW, 1999. Overexpression of hypoxia-inducible factor 1alpha in common human cancers and their metastases. Cancer Res 59: 5830–5835. 10582706

[12] Abulizi A , 2019. *Echinococcus multilocularis* inoculation induces NK cell functional decrease through high expression of NKG2A in C57BL/6 mice. BMC Infect Dis 19: 792. 31500589 10.1186/s12879-019-4417-1 PMC6734356

[13] Grüner B Kern P Mayer B Gräter T Hillenbrand A Barth TEF Muche R Henne-Bruns D Kratzer W Kern P, 2017. Comprehensive diagnosis and treatment of alveolar echinococcosis: a single-center, long-term observational study of 312 patients in Germany. GMS Infect Dis 5: Doc01. 30671323 10.3205/id000027 PMC6301735

[14] Tuxun T Apaer S Ma HZ Zhao JM Lin RY Aji T Shao YM Wen H, 2018. Plasma IL-23 and IL-5 as surrogate markers of lesion metabolic activity in patients with hepatic alveolar echinococcosis. Sci Rep 8: 4417. 29535327 10.1038/s41598-018-20301-8 PMC5849767

[15] Schwarze V Mueller-Peltzer K Negrão de Figueiredo G Lindner F Rübenthaler J Clevert DA, 2018. The use of contrast-enhanced ultrasound (CEUS) for the diagnostic evaluation of hepatic echinococcosis. Clin Hemorheol Microcirc 70: 449–455. 30347607 10.3233/CH-189310

[16] Wa ZC Du T Li XF Xu HQ Suo-Ang QC Chen LD Hu HT Wang W Lu MD, 2020. Differential diagnosis between hepatic alveolar echinococcosis and intrahepatic cholangiocarcinoma with conventional ultrasound and contrast-enhanced ultrasound. BMC Med Imaging 20: 101. 32854653 10.1186/s12880-020-00499-8 PMC7453544

[17] Li J Dong J Yang L Li X Song T, 2018. Comparison of [(18)F]fluorodeoxyglucose positron emission tomography and contrast-enhanced ultrasound for evaluation of hepatic alveolar echinococcosis activity. Ultrasound Med Biol 44: 2199–2208. 30077410 10.1016/j.ultrasmedbio.2018.06.010

[18] Hanahan D Weinberg RA, 2011. Hallmarks of cancer: the next generation. Cell 144: 646–674. 21376230 10.1016/j.cell.2011.02.013

[19] Svagzdys S Lesauskaite V Pavalkis D Nedzelskiene I Pranys D Tamelis A, 2009. Microvessel density as new prognostic marker after radiotherapy in rectal cancer. BMC Cancer 9: 95. 19323831 10.1186/1471-2407-9-95 PMC2666763

